# Advanced Photocatalytic Treatment of Wastewater Using Immobilized Titanium Dioxide as a Photocatalyst in a Pilot-Scale Reactor: Process Intensification

**DOI:** 10.3390/ma15134547

**Published:** 2022-06-28

**Authors:** Abdoulaye Kane, Achraf Amir Assadi, Atef El Jery, Ahmad K. Badawi, Hamza Kenfoud, Oussama Baaloudj, Aymen Amin Assadi

**Affiliations:** 1 UniLaSalle-Ecole des Métiers de l’Environnement, Cyclann, Campus de Ker Lann, 35170 Bruz, France; 2 Research Unit Advanced Materials, Applied Mechanics, Innovative Processes and Environment, Higher Institute of Applied Sciences and Technology of Gabes (ISSAT), University of Gabes, Sfax 3018, Tunisia; achraf.assadi@gmail.com; 3 Department of Chemical Engineering, College of Engineering, King Khalid University, Abha 61411, Saudi Arabia; ajery@kku.edu.sa; 4 Civil Engineering Department, El-Madina Higher Institute for Engineering and Technology, Giza 12588, Egypt; dr.ahmedkaram91@gmail.com; 5 Laboratory of Reaction Engineering, Faculty of Mechanical Engineering and Process Engineering, USTHB, BP 32, Algiers 16111, Algeria; hamza.kenfoud.93@gmail.com (H.K.); obaaloudj@gmail.com (O.B.); 6 Univ. Rennes, École Nationale Supérieure de Chimie de Rennes, CNRS, ISCR (Institut des Sciences Chimiques de Rennes)–UMR 6226, av. du Général Leclerc, 35700 Rennes, France

**Keywords:** pilot-scale treatment, photocatalysis, intensification, antibiotics, flumequine

## Abstract

In many nations, particularly those experiencing water scarcity, novel approaches are being applied to clean wastewater. Heterogeneous photocatalysis is the most widely used of these approaches because it entails the decomposition of organic molecules into water and carbon dioxide, which is a more ecologically benign process. In our study, we studied the photocatalytic degradation process on the effluent flumequine. This treatment is made through a solar pilot reactor in the presence of immobilized titanium dioxide with three light intensities and two types of water as solvents. A variety of factors that might influence the rate of deterioration, such as flow rate, light intensity, and initial concentration, have been investigated. The maximal degradation of flumequine was achieved at more than 90% after 2.5 h under optimal conditions (an initial concentration of 5 mg/L, three lamp light intensities, and a flow rate of 29 L/h). By combining the oxidized agent H_2_O_2_ with this process, the photocatalytic activity was improved further to 97% under the same conditions. The mineralization of this product has also been tested using total organic carbon (TOC) analysis. A high mineralization rate has been recorded at around 50% for a high initial concentration (20 mg/L) at a flow rate of 126 L/h. The results demonstrated the highly effective removal of flumequine and the efficacy of this photocatalytic system.

## 1. Introduction

The framework law on water (Water Framework Directive, WFD) and the Wastewater Systems Effluent Regulations (WSER) impose a discharge threshold for several substances [1]. Thus, Directive 2000/60/EC of 23 October 2000 (WFD) establishes objectives for the protection and restoration of surface water quality (freshwater, coastal waters, and groundwater) [2]. It has also strengthened the protection of the aquatic environment through measures to progressively reduce discharges, emissions, and losses of hazardous or priority substances into the water [3]. This regulation, therefore, applies to health care institutions, including hospitals. The latter is required to comply with threshold values and to limit the number of hazardous substances discharged into the network [4]. The general objective of the WFD is to achieve good status in the various environments throughout Europe by 2021 [5]. The treatment of hospital effluents cannot be neglected due to the large volume of water entering hospitals and being discharged into sewerage systems, generating environmental pollution [4,6]. Hospital effluents are specific to health establishments; they are mainly generated by the activities of care, analysis, and research [7,8]. Within the framework of this study, we will be working on the effluents of the Chu de Rennes (a capacity of 10,000 inhabitants in waste equivalent), investigating the question of the origin of various micropollutant substances within the Research of Dangerous Substances in Water (RDSW) framework. The major problems posed by these micropollutants is their toxicity, that is, the carcinogenic, mutagenic, reprotoxic (CMR) character of some of them [9]. Because of this toxicity, they have an impact on the environment (animals and plants) as well as on humans [10]. Moreover, they have an indirect impact on humans and the environment because of the disruption of natural cycles [11].

To date, there are several relevant technologies listed by the INERIS (French National Competence Center for Industrial Safety and Environmental Protection), such as pre-chlorination, coagulation, flocculation and decantation methods, ion exchange, adsorption on activated carbon, membrane filter, etc. [12,13,14,15]. These techniques offer a solution to this problem [16]. Among these, photocatalysis and the coupling of photocatalysis and strong oxidants (ozone, hydrogen peroxide, etc.) show interesting perspectives in terms of the degradation/mineralization of hospital effluents, with low energy consumption [17,18]. Hospitals have a high level of water consumption; they need nearly 1000 L of water per day and per bed, which is 10 times higher than the daily consumption per local inhabitant. This water consumption leads to significant discharges [19].

These discharges are often loaded with microorganisms, antibiotics, toxic chemicals, antiseptics, and detergents. This makes them complex mixtures. In these effluents, there are three types of sources, mainly: (i) effluents from clinical services, (ii) effluents from medico-technical services, and (iii) effluents resulting from the maintenance of the premises and machines.

In the effluents from clinical services, many compounds have been found, for example: betadine, which is an iodized compound; chlorhexidine, which is a chlorinated compound; and aldehyde forms such as glutaraldehyde, which is a molecule that is toxic to humans and the environment. Traces of mercury are present in the thermometers of various radiology services, as well as the residues of their medicines. These discharges are all different types and are present under diverse concentrations [20].

The effluents produced by medical–technical services are composed of biological liquids and pathogenic germs that are viral and/or parasitic; they can be evacuated in black water and with the products of analysis used in laboratories if no specific system of treatment or recovery is set up [21]. In the effluents resulting from the maintenance of premises and machines, there is the presence of bleach, various disinfectants, etc. In fact, all these different compounds do not have similar characteristics, although they may come from the same department. The problem raised by large discharges of hospital effluents is that they are discharged into municipal sewage systems without prior treatment and cause saturation effects, which can lead to a release of pollutants into the natural environment. In fact, organochlorine compounds, mercury, and drug residues undergo little or no degradation when leaving treatment plants [22].

The majority of traditional wastewater treatment plants are not intended to remove the contaminants present in hospital effluents. Therefore, suitable and economically feasible techniques must be developed and applied to reduce hospital discharges into the environment. Physicochemical methods, such as membrane filtration [23] and activated carbon adsorption [24], have been applied to remove pharmaceutical contaminants [25,26]. However, the main disadvantage of these methods is that they do not eliminate (nor mineralize) the pollutants, but rather, they transfer them from one phase to another [3]. Nowadays, advanced oxidation processes (AOPs) have been proposed as alternative methods for the removal of pollutants in hospital effluents among other compounds in wastewater [27,28]. The principle of advanced oxidation processes (O_3_/H_2_O_2_, UV/O_3_, UV/H_2_O_2_, H_2_O_2_/Fe^2+^, and UV-TiO_2_) is to produce a hydroxyl radical in water, which is a very powerful oxidant (E° = 2.8 V). It is capable of oxidizing a wide range of organic compounds with one or more double bonds. As reported by Homem and Santos (2011), hydroxyl radicals are produced from oxidizing agents, such as ozone and hydrogen peroxide, and are often combined with UV radiation or semiconductor/metal oxide catalysts [29]. In recent years, several reactors have been developed on a pilot scale to meet the needs of wastewater treatment. In fact, Rabahi et al. [30] investigated the photocatalytic treatment of petroleum industry wastewater using a recirculating annular reactor at a flow rate of 1 m^3^/h. Indeed, work on TiO_2_ deposited on luminous textiles has been carried out. The TiO_2_ support is illuminated by a UV LED emitter connected to a degraded optical fiber. The good efficiency of TiO_2_ on luminous textile in the elimination of flumequine in water was demonstrated: During this study, two configurations of luminous textiles were used (single-sided and double-sided), and it was proven that the photocatalytic performance of the double-sided was better compared to the single-sided in ultrapure water [31].

This study aims to propose an advanced, photocatalytic, pilot-scale reactor in order to treat pharmaceutical contaminants that contain immobilized titanium dioxide as a photocatalyst. A pilot-scale reactor was described, along with its operating parameters, in detail. The immobilized catalyst based on TiO_2_ deposited on cellulosic paper was characterized. Moreover, the kinetics of antibiotics were investigated using a semiconductor photocatalyst irradiated by UV light sources. The effect of key operating parameters, such as initial concentration and flow rate, was also investigated. Furthermore, the kinetic studies were intensified by adding an oxidant.

## 2. Materials and Methods

### 2.1. Catalyst and Reactor

In this research, the catalyst was manufactured and supplied by the Ahlstrom company. It consists of cellulose paper with TiO_2_ nanoparticles deposited on it (cellulose paper + 10 g/m² of colloidal silica + 10 g/m² of TiO_2_ P25-Degussa). Titanium dioxide was deposited on organic tissue via impregnation using an industrial-sized press. A dry mixture of 50 wt.% colloidal silica and 50 wt.% titanium dioxide nanoparticles (P25-Degussa) were suspended in pure water. In order to ensure the deposition of the dry TiO_2_ on tissue support, the suspension was composed of 40% dry powder and 60% pure water. The press was employed to impregnate fibers with the suspension; then, they were dried.

This catalysis exhibited chemical stability, validated based on our previous work on the photocatalytic treatment of petroleum industry wastewater using a recirculating annular reactor [30].

**Figure 1:**
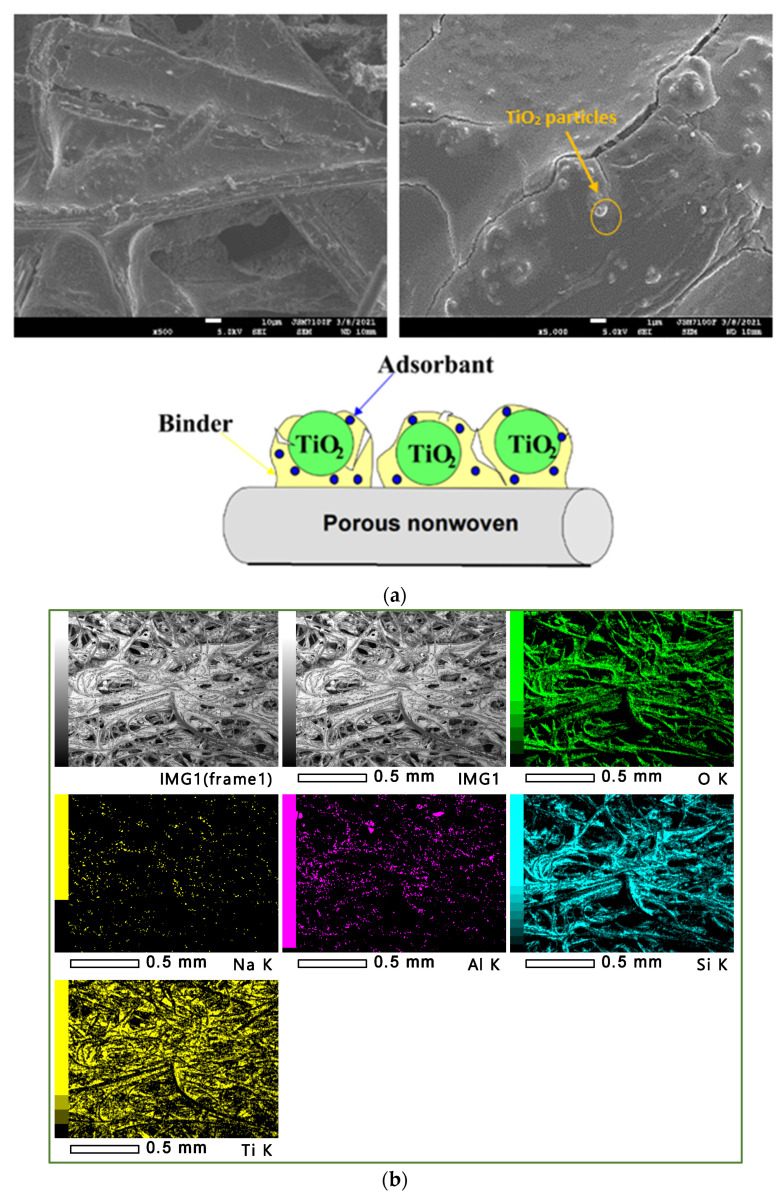
(**a**) Catalyst images under a scanning electron microscope, SEM (×500 and ×5000 as magnification ratios), with an illustrative presentation of the suspended TiO_2_ nanoparticles. (**b**) Catalyst images, SEM/EDX, with an illustrative presentation of the suspended TiO_2_ nanoparticles.

Figure 1 displays the scanning electron microscope characterization of the catalyst and an illustrative presentation of the TiO_2_ nanoparticles that were suspended and deposited on the paper.

The SEM/EDX analysis provided an enlarged view of the catalyst sample, showing the different elements of the TiO_2_ deposited on the cellulosic paper, such as: titanium, sodium, oxygen, aluminum, and silicon corresponded to the silica deposit. Moreover, Figure 1b shows a uniform Ti distribution in the cellulosic paper.

**Figure 2:**
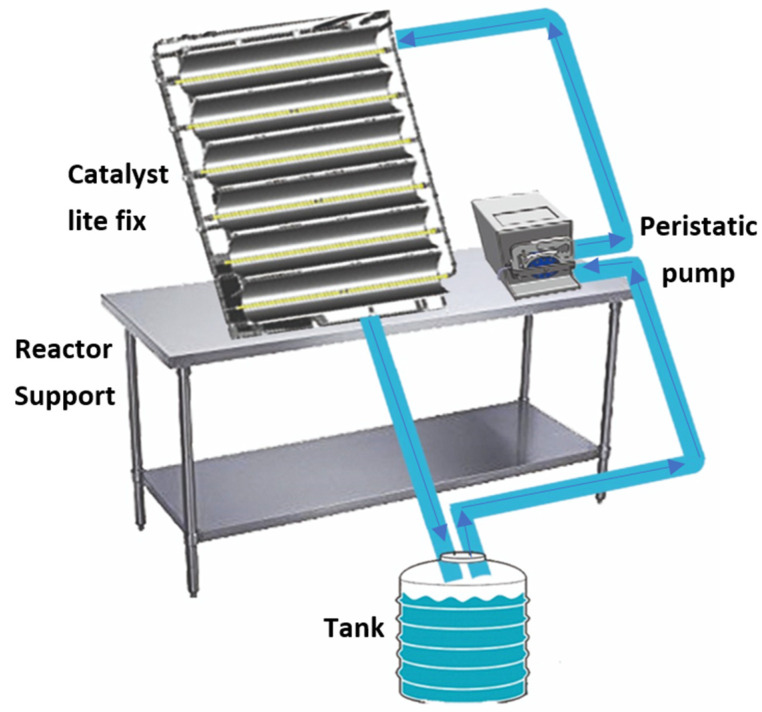
Operating diagram of the staircase reactor.

The device used in the study was developed by the Ahlstrom company. It mainly consists of a reactor with a falling film flow along a staircase (see Figure 2). A falling film was formed during the flow of the effluent to be treated. The untreated liquid flowed horizontally. This type of reactor has advantages such as:

- A thin layer of effluent (film thickness of about ~1 mm), which reduces the absorption of UV rays by the untreated effluent.
- The step configuration of the reactor increases the exchange surface between the catalyst and the liquid film. In the same way, the exchange surface of the effluent with the air is increased, thus improving the transfer of oxygen into the water and improving oxidation.

The reactor also consisted of Philips UV lamps (PL-L 24W/10/4P) with a maximum wavelength of 365 nm.

- A 2 L tank with a magnetic agitator for the effluent recirculation.
- A peristaltic pump with a maximum flow rate of 150 L/h.

### 2.2. Pollutants

Flumequine (98%) and nonylphenol (PESTANAL^®^, analytical standard) are products sold by Sigma-Aldrich (St. Louis, MO, USA). Flumequine is an antibiotic molecule of the quinolone class. It inhibits the enzymatic action of bacterial DNA gyrases. Nonylphenol is a synthetic organic compound belonging to the alkylphenol family. It is used as a precursor in the manufacture of polyethoxylated nonylphenols.

### 2.3. Analysis Methods

A Varian Cary 50 probe spectrophotometer (Varian, The Netherlands) was used to measure the absorbance of flumequine in the effluent. The spectrophotometer produces a wavelength range of 190 and 1100 nm, and the peak of flumequine adsorption is 246 nm. For sample preparation, 3 mL of effluent was taken and filtered at 0.45 µm. The filtered solution was put in a 10 mm Hellma quartz tank. TOC meter TOC-V Series TOC analyzers from Shimadzu (Tokyo, Japan) were used in order to measure the mineralization rate. An amount equal to 40 mL of solution was taken at every hour of degradation as samples to be analyzed. Then, the solution was filtered by a 0.45 µm filter.

## 3. Results and Discussion

Before each experiment, the UV was turned off and then, once the inlet and outlet concentrations of the pollutant were the same (adsorption equilibrium state), the reactor power (UV light) was turned on. Output samples were collected at 30- to 60-min intervals until a steady state was achieved. At the end of the experiment, the reactor was cleaned with a flow of clean water for at least 1 h.

### 3.1. Initial Concentration Effect on Photocatalytic Reaction

**Figure 3:**
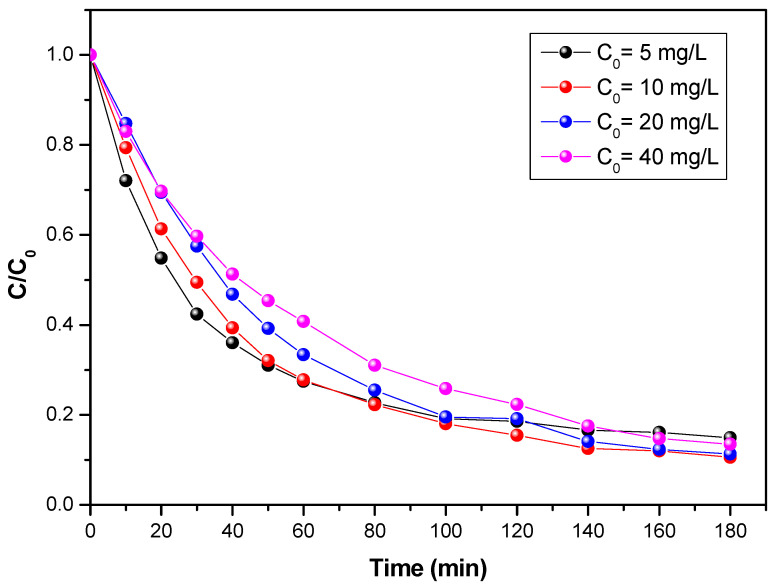
Evolution of the concentration of flumequine during its photocatalytic degradation versus time for four initial concentrations at a flow rate of 29 L/h and a UV of 38 W/m^2^.

A representation of the evolution of the concentration as a function of time for four different initial concentrations is shown in Figure 3. It can be observed that the increase in the concentration globally disadvantages the degradation of the flumequine pollutant. At low concentrations, the kinetics are faster, which can be explained by the fact that more molecules are “available” in the solution, and UV light reaches the catalyst surface more easily, leading to an increase in the photocatalytic degradation rate. However, at higher concentrations, the molecules start to act as a filter for the incident UV light, so that the light hardly reaches the TiO_2_ surface, leading to a slowdown of photocatalytic degradation. This same trend was observed by Sharma et al. 2010 [32]. The photodegradation mechanism was proposed as [33]:

(1) $${\mathrm{TiO}}_{2}+h\upsilon \;\to {\;\mathrm{TiO}}_{2}{}^{*}+e^{-}+h^{+}$$

(2) $$H_{2}O+h^{+}\;\to {\;\mathrm{OH}}^{*}+H^{+}$$

(3) $$O_{2}+e^{-}\to {\;O}_{2}{}^{-*}$$

(4) $$\begin{array}{l} \;\mathrm{Pollutant}+{\mathrm{OH}}^{*}{\;\mathrm{and}\;O}_{2}{}^{-*}\\ \;\;\;\;\to {\mathrm{CO}}_{2}+H_{2}O\;+\mathrm{intermediate}\;\mathrm{by}\;\mathrm{products} \end{array}$$

### 3.2. Influence of Flow Rate

**Figure 4:**
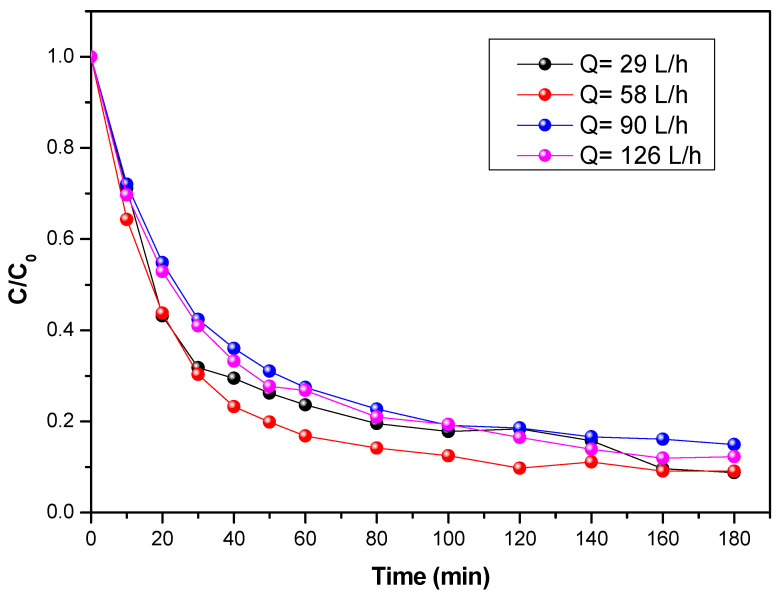
Evolution of the concentration of flumequine during its photocatalytic degradation for four flow rates at an initial concentration of 5 mg/L and a UV of 38 W/m^2^.

The limitation of mass transfer is a determining factor when studying a continuous photocatalytic process. This mass transfer limitation can lead to a low degradation rate. From a hydrodynamic point of view, a reaction involving a catalytic solid can be limited by its external mass transfer. Looking at Figure 4, it seems that the degradation kinetics are less efficient at high flow rates and at low flow rates. There would be an optimal flow rate for which a faster degradation would be obtained. Several experiments with different initial concentrations were performed on the staircase reactor for flumequine removal. For each experiment, the initial velocity was determined by calculating the slope of the C = f(t) curve over the first 30 min. The representation of the initial velocity as a function of the initial concentration for four different flow rates is shown in Figure 4.

By observing Figure 4, the increase in the initial rate of degradation in the flumequine with the initial concentration can be seen. When the concentration is low, the initial speed is directly proportional to the initial concentration (reaction of order 1). However, for a certain value of the concentration, the speed of degradation tends toward a maximum (order 0).

Indeed, the evolution of a kinetic of order 1 toward an order 0 has been widely described in the literature. Several hypotheses have been formulated to explain this behavior [34,35]:

- At a certain concentration, all adsorption sites are occupied and an increase in the initial concentration has no significant influence on the degradation rate.
- The photogenerated electron–hole pairs are formed first, followed by their interaction with organic molecules. The kinetically limiting step varies depending on the pollutant concentration: At low concentrations, the chemical reaction (oxidation of the organic pollutant) is limiting, but at high concentrations, the production of photogenerated species becomes the kinetically deciding phase.
- The degradation rate of the parent chemical is influenced by the intermediates generated during the photocatalytic process. At high pollutant concentrations, the amount of byproducts generated is greater, limiting pollutant breakdown even more. From these values of initial velocities, through the relationship between 1/r and 1/C in Equation (5) (obtained by linearization of the Langmuir–Hinshelwood isotherm), we calculate the kinetic constants, k, and the equilibrium constants, K, hereafter, the linearized Langmuir–Hinshelwood isotherm.

(5) $$\frac{1}{r}=\frac{1}{\mathrm{kKC}}+\frac{1}{k}$$

The layout of 1/r_0_ = f(1/C_0_) for each flow rate (29 L/h, 58 L/h, 90 L/h, and 126 L/h) provides access to the values of k and K, recorded and listed in Table 1.

The variation of k values versus flow rate shows that the degradation kinetics increases in a nonlinear way with the flow rate, reaching a maximum at 58 L/h before decreasing to reach a tray. Indeed, at this range of flowrate (29–58 L/h), an increase in flowrate can generate a turbulence zone at the catalytic surface, which leads to improving the mass transfer step of the pollutant from the bulk to solid phase.

There is an optimal flow rate at which the degradation of flumequine is higher. It could be explained as follows:

- For low flow rates, the residence time was higher compared to high flow rates. The flumequine pollutant had enough time to react with the radicals in the interface of the solution and the catalytic surface. In addition, the thickness of the water film is thinner. Oxygen is much more accessible to contact the catalytic surface. Moreover, a thin water film contributes to obtaining a sufficient light intensity.
- For the highest flow rates, low kinetic constants were observed. Indeed, by increasing the flow rate, the residence time decreased and the thickness of the water film increased, which had the disadvantage of preventing photons from entering the interface of the two phases (liquid/solid = catalyst).

Moreover, according to the work of Behnaz Sheidaei et al. 2016 [36], the apparent kinetic constant of K increases proportionally with the volume flow rate.

Similarly, the increase in K with flow rate may be related to more liquid flowing through the photocatalytic reactor and the increased enhancement of material transfer; this observation was made by Hao et al. 2009 [37].

Al-Ekabi et al. [38] conducted phenol degradation experiments on two distinct reactors (each reactor was studied in multi-pass and single-pass mode). In multi-pass mode, they observed that, for the two distinct reactors, the degradation rates were higher at high flow rates. On the contrary, with the single-pass mode, the best degradation rates were observed at low flow rates. This teaches us that the effects of the flow rate also depend on the configuration/architecture of the reactor.

### 3.3. Solvent Effect on Photocatalytic Performances (Ultrapure Water/Tap Water)

**Figure 5:**
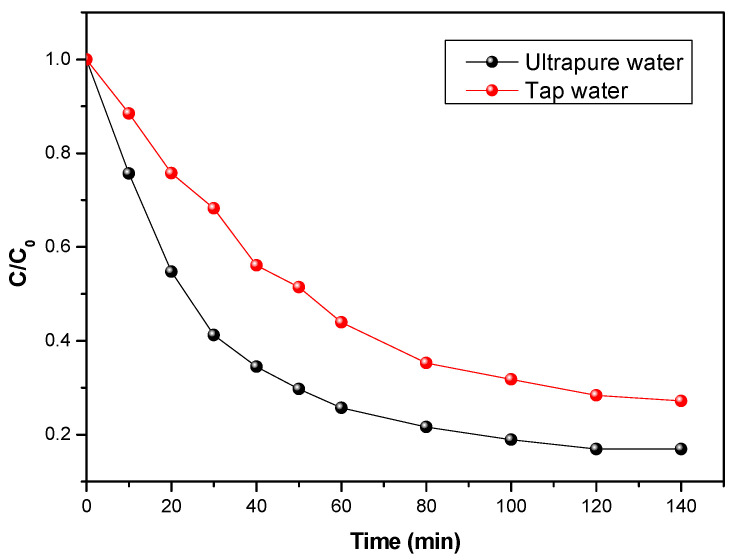
Evolution of the concentration of flumequine: Comparison between tap water and ultrapure water as solvents to induce degradation, with an initial concentration of 5 mg/L, UV 38 W/m^2^, and a flow rate of 126 L/h.

To better study the treatment of hospital effluents, a comparison was made between effluents prepared from tap water and ultrapure water. The manipulations were performed with flumequine at 5 mg/L and for a volume flow rate of 126 L/h. The evolution of flumequine concentration versus time is presented in Figure 5. It can be observed that, for the effluent prepared with EUP, the degradation rate of flumequine was faster than in the case of the effluent prepared with tap water.

This behavior can be explained by the presence of ions or other molecules (chloride ions, nitrates, iron, etc.) in the tap water. Indeed, the presence of cations and soluble organic matter in real waters generates competition on the active catalyst sites. The different molecules present in the tap water can react as inhibitors of the photocatalytic reaction, which has the effect of directly influencing the degradation rate. This question is important to address in this study, since we will encounter these degradation inhibitors during the treatment of hospital effluents.

### 3.4. Effect of UV Intensity

**Figure 6:**
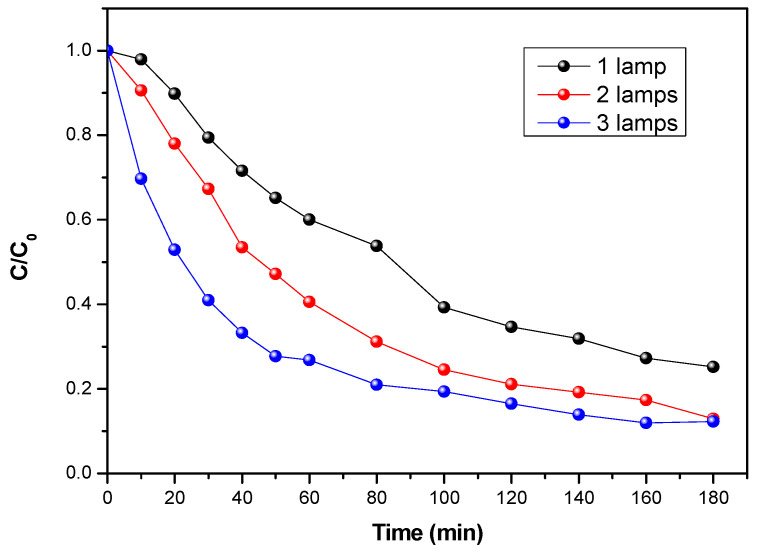
Evolution of the concentration of flumequine during its photocatalytic degradation for an initial concentration of 5 mg/L, three light intensities, and a flow rate of 29 L/h.

To determine the impact of UV intensity on flumequine breakdown and mineralization, experiments at different UV intensities were performed with flumequine in the photocatalytic reactor. Different light intensities were applied by varying the number of lamps: one lamp emitted a UV intensity of 14.5 W/m^2^, two lamps emitted a UV intensity of 27 W/m^2^, and three lamps emitted a UV intensity of 38 W/m^2^. Here, the flow rate and the pH value were kept constant at 29 L/h and 6.6 L/h, respectively. Light intensity is an elementary factor in photocatalytic degradation, as electron–hole pairs are produced by UV light energy. Figure 6 shows that the degradation kinetics of flumequine are faster with an increasing light intensity for a fixed initial concentration. This is explained by the fact that, by increasing the light intensity, more energy reaches the surface of the TiO_2_ for a better production of electron–hole pairs, which achieves better degradation efficiencies. It is therefore obvious that the kinetic constant, K, increases with light intensity.

### 3.5. Mineralization during the Degradation of Flumiquine

**Figure 7:**
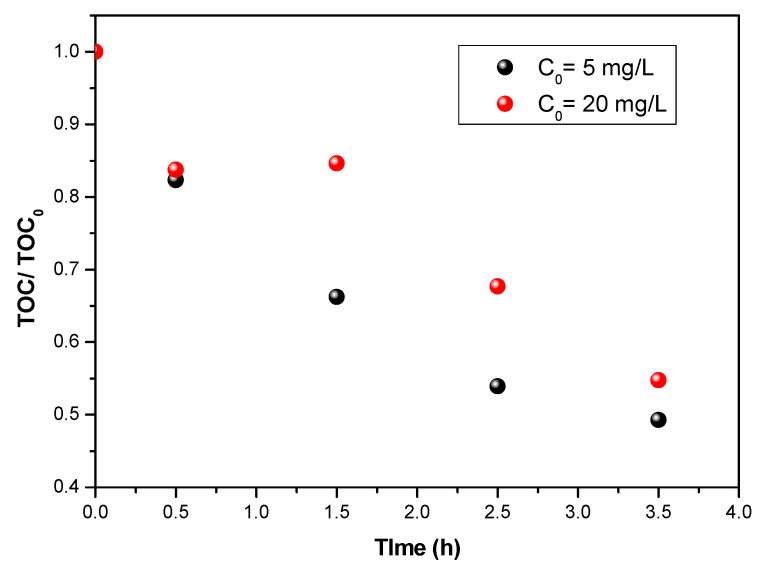
TOC evaluation during flumequine degradation for two initial concentrations for UV 38 W/m^2^ and a flow rate of 126 L/h.

Knowledge about the degradation efficiency of the substance to be treated is important, but it is not sufficient to judge the quality of the decontamination. It is essential to mineralize the initial molecules and their byproducts in order to ensure the best possible decontamination. The evolution of mineralization for flumequine was determined by measuring TOC as a function of time. By observing Figure 7, it can be seen that, during the first 30 min, the evolution of the TOC is identical for the two initial concentrations. The TOC value then decreased more slowly for a low initial concentration (5 mg/L) compared to an initial concentration of 10 mg/L.

**Figure 8:**
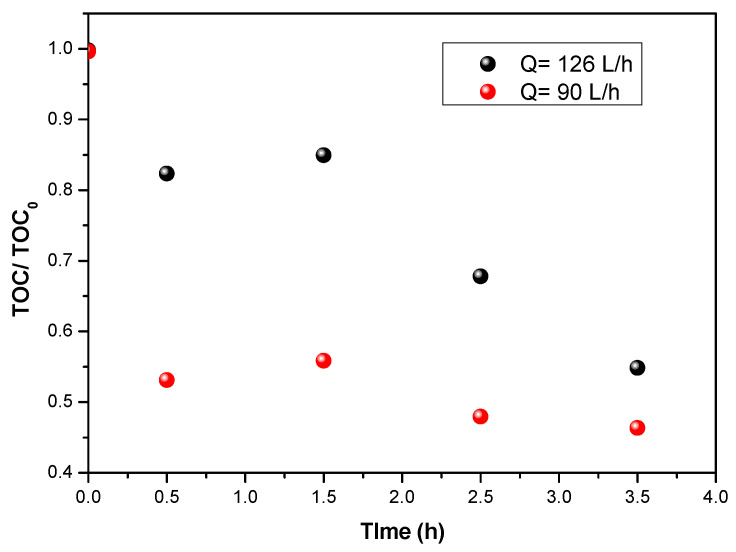
Evaluation of TOC during the degradation of flumequine for two flow rates (90 L/h and 126 L/h) at an initial concentration of 5 mg/L and UV of 38 W/m^2^.

Figure 8 clearly shows that, for a fixed initial flumequine concentration (5 mg/L), better mineralization is obtained at 90 L/h than at 126 L/h. As mentioned above, this could be explained by the fact that, at high flow rates, the residence time decreases, and the thickness of the water film tends to increase, the consequence being that few photons will be able to reach the catalyst/solution interface.

### 3.6. Enhancing Photocatalytic Activity by Coupling Photocatalysis and Hydrogen Peroxide (H_2_O_2_)

**Figure 9:**
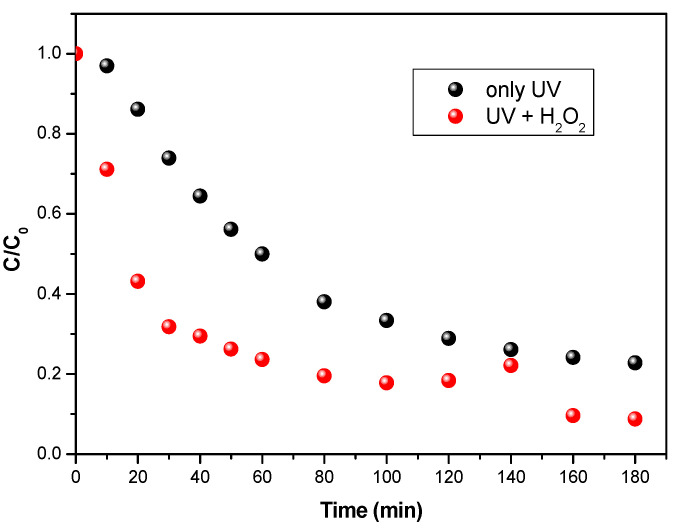
Evolution of the concentration of flumequine: comparison between UV alone and coupling UV + H_2_O_2_. C_0 flumequine_ = 5 mgl/L and a flow of 29 L/h. n_0_H_2_O_2_ = 1 µmol.

It is now well known that photocatalytic processes can degrade certain compounds, but that they have a potentially low mineralization capacity and, consequently, a tendency to form byproducts or intermediate compounds. One way to improve the efficiency of the photocatalytic process is to add hydrogen peroxide to the reaction medium, leading to a photolysis of the latter and giving rise to the formation of hydroxyl radicals at controllable rates and concentrations. It is in this respect that the influence of H_2_O_2_ on photocatalysis is examined in this study. For this purpose, 200 µL of a hydrogen peroxide solution (C_H2O2_ = 1 µmol/L) was added to the initial solution to be treated (C_0 Fluméquine_ = 5 mgl/L). Figure 9 shows the degradation kinetics of flumequine (with and without the addition of H_2_O_2_) and the initial rates observed. The addition of H_2_O_2_ to the reaction medium accelerated the photocatalytic reaction. For an irradiation time of 180 min, the abatement achieved with a solution of an initial concentration of 5 mg/L in flumequine is 71% without the addition of H_2_O_2_, while this value increases to 91% in the presence of H_2_O_2_. Moreover, we noted an increase in the initial degradation rate from 3.5 × 10^−2^ to 9 × 10^−2^ mg/L min. These results were confirmed by a faster degradation (higher initial speed) in the presence of H_2_O_2_.

## 4. Conclusions

In our work, we investigated the photocatalytic degradation process of flumequine with a step reactor. This treatment was conducted in a solar pilot reactor in the presence of immobilized titanium dioxide with three light intensities and two types of water as solvents. We tested the different factors that could influence the degradation rate, such as flow rate, light intensity, and initial concentration. Under optimal conditions (an initial concentration of 5 mg/L, three lamp light intensities, and a flow rate of 29 L/h), the maximum degradation of flumequine was achieved at more than 90% after 2.5 h. Moreover, we intensified the photocatalytic processes by combining H_2_O_2_ oxidizing agents, and the photocatalytic activity was boosted even further to 97% under the same conditions. This product’s mineralization was also examined using TOC, and a high mineralization rate was discovered around 50% for a high initial concentration (20 mg/L) and at a flow rate of 126 L/h. We studied the flow rate effect using the isotherm Langmuir–Hinshelwood model. The results showed that flumequine was removed extremely efficiently, almost completely, demonstrating the efficacy of this pilot system.

## Figures and Tables

**Table 1:** Values of k(mg/L·min) and K (L·mg) for different flow rates.

Flow Rates	29 L/h	58 L/h	90 L/h	126 L/h
k (mg/L·min)	1.09	1.49	0.55	0.56
K (L·mg)	1.81 × 10^–2^	1.09 × 10^–2^	3.14 × 10^–2^	2.48 × 10^–2^

## Data Availability

Not applicable.
